# Quine in Spasms with Hooping Cough

**Published:** 1872-06

**Authors:** Thos. H. Mayo

**Affiliations:** Columbus, Miss.


### QUININE IN SPASMS WITH HOOPING COUGH.

BY THOS. H. MAYO, M. D , OF COLUMBUS, MISS.

At 5 P. M., January 19, 1872, I was called to see a child seventeen months of age, Found the little patient in spasms, which had lasted one half hour. Child was large and well nourished; was in the fourth week of hooping cough ; had some fever every day for several days; vomited freely when it coughed, and for the last few days seemed languid and weak.

Pulse small and frequent; pupils somewhat dilated, but little affected by light; did not recognize any one ; winked but seldom ; eyes remained wider open than was natural; a constant moaning ; mucous rales; breathing irregular; limbs extended and so rigid that the patient could not be put into an ordinary tub for a hot bath.

The tr. assfœtida dr. j. had been given before my arrival and chloroform was being administered by inhalation. I continued the chloroform, and gave by enema (the patient could not swallow) tr. assafœtida, tr. lobelia a a dr. i. and repeated it in one half hour. Applied sinapisms to the whole length of the spine; waited one hour ; no effect from remedies.

Then gave morphia acetas gr. ⅛., repeated the dose in one half hour; friction and chloroform continued until 9 P. M.; no improvement; rigidity continues, with some nervous twitching every few minutes; two or three paroxysms of coughing have occurred in the meantime.

I then decided to try quinine in large doses, as suggested by Dr. Maxwell, of this city: gave 10 gr. in starch water by enema. In fifteen minutes the knees lost their rigidity, and in less than half an hour the patient was completely relaxed, and the spasm had gone off. Patient continued to moan, eyes wide open, until 11 P. M.; then had a prolonged and severe spasm of the diaphragm; respiration stopped, and pulse not perceptible for one or two minutes. I resorted to artificial respiration; patient soon revived and seemed to improve.

I then applied a blister to the chest; continued wakefulness; moaned less. Gave two doses, 5 gr. each, of hydrate of chloral, which produced a calm sleep of several hours. Patient had no return of spasm; convalescence was steady but protracted.

In order to further test the efficacy of quinine in spasms, I recommend its use in this form of disease which we are so frequently called upon to treat in general practice.

It will be observed that in this case antispasmodics and chloroform produced no effect.

